# Analysis of Parent-of-Origin Effects on the X Chromosome in Asian and European Orofacial Cleft Triads Identifies Associations with *DMD, FGF13, EGFL6*, and Additional Loci at Xp22.2

**DOI:** 10.3389/fgene.2018.00025

**Published:** 2018-02-22

**Authors:** Øivind Skare, Rolv T. Lie, Øystein A. Haaland, Miriam Gjerdevik, Julia Romanowska, Håkon K. Gjessing, Astanand Jugessur

**Affiliations:** ^1^Department of Occupational Medicine and Epidemiology, National Institute of Occupational Health, Oslo, Norway; ^2^Department of Global Public Health and Primary Care, University of Bergen, Bergen, Norway; ^3^Centre for Fertility and Health (CeFH), Norwegian Institute of Public Health, Oslo, Norway; ^4^Department of Genetics and Bioinformatics, Norwegian Institute of Public Health, Oslo, Norway; ^5^Computational Biology Unit, University of Bergen, Bergen, Norway

**Keywords:** parent-of-origin, X chromosome, GWAS, case-parent triads, orofacial clefts, birth defects, genetic epidemiology, Haplin

## Abstract

**Background:** Although both the mother's and father's alleles are present in the offspring, they may not operate at the same level. These parent-of-origin (PoO) effects have not yet been explored on the X chromosome, which motivated us to develop new methods for detecting such effects. Orofacial clefts (OFCs) exhibit sex-specific differences in prevalence and are examples of traits where a search for various types of effects on the X chromosome might be relevant.

**Materials and Methods:** We upgraded our R-package Haplin to enable genome-wide analyses of PoO effects, as well as power simulations for different statistical models. 14,486 X-chromosome SNPs in 1,291 Asian and 1,118 European case-parent triads of isolated OFCs were available from a previous GWAS. For each ethnicity, cleft lip with or without cleft palate (CL/P) and cleft palate only (CPO) were analyzed separately using two X-inactivation models and a sliding-window approach to haplotype analysis. In addition, we performed analyses restricted to female offspring.

**Results:** Associations were identified in “Dystrophin” (*DMD*, Xp21.2-p21.1), “Fibroblast growth factor 13” (*FGF13*, Xq26.3-q27.1) and “EGF-like domain multiple 6” (*EGFL6*, Xp22.2), with biologically plausible links to OFCs. Unlike *EGFL6*, the other associations on chromosomal region Xp22.2 had no apparent connections to OFCs. However, the Xp22.2 region itself is of potential interest because it contains genes for clefting syndromes [for example, “Oral-facial-digital syndrome 1” (*OFD1*) and “Midline 1” (*MID1*)]. Overall, the identified associations were highly specific for ethnicity, cleft subtype and X-inactivation model, except for *DMD* in which associations were identified in both CPO and CL/P, in the model with X-inactivation and in Europeans only.

**Discussion/Conclusion:** The specificity of the associations for ethnicity, cleft subtype and X-inactivation model underscores the utility of conducting subanalyses, despite the ensuing need to adjust for additional multiple testing. Further investigations are needed to confirm the associations with *DMD, EGF16*, and *FGF13*. Furthermore, chromosomal region Xp22.2 appears to be a hotspot for genes implicated in clefting syndromes and thus constitutes an exciting direction to pursue in future OFCs research. More generally, the new methods presented here are readily adaptable to the study of X-linked PoO effects in other outcomes that use a family-based design.

## Introduction

Orofacial clefts (OFCs) are the most common craniofacial birth defects in humans, affecting approximately 1–2/1000 live births worldwide (Mossey et al., 2009). They are broadly categorized according to whether the affected region includes the primary palate, the secondary palate, or both, and whether they occur with or without additional congenital anomalies (Dixon et al., 2011; Marazita, 2012; Beaty et al., 2016). OFCs pose a substantial public health burden in terms of the medical costs and sequelae associated with their treatment, which may persist from infancy to childhood and throughout life (Wehby and Cassell, 2010; Berg et al., 2016a,b). OFCs have also been linked to higher risk of specific types of cancer in later life (Zhu et al., 2002; Bille et al., 2005), increased overall mortality well into adulthood (Christensen et al., 2004), and lower academic achievement (Wehby et al., 2014). Despite major progress in surgery and other medical interventions aimed at repairing and managing the cleft itself, current understanding of the biological underpinnings of these relatively common birth defects is still incomplete.

Both genetic and environmental factors have been reported to influence the risk of OFCs, either individually or through their complex interactions in relevant biological pathways (Jugessur et al., 2009a; Dixon et al., 2011; Marazita, 2012; Rahimov et al., 2012; Beaty et al., 2016; Kousa and Schutte, 2016). Genome-wide association studies (GWAS) have successfully identified several genes and loci for OFCs, contributing to an improved understanding of the biological processes underlying these relatively common birth defects (Birnbaum et al., 2009; Grant et al., 2009; Beaty et al., 2010; Mangold et al., 2010; Camargo et al., 2012; Ludwig et al., 2012; Wolf et al., 2015; Leslie et al., 2016a,c). However, most of the genetic variation in OFCs remains unexplained. Given the more than 30-fold increased risk of recurrence in first-degree relatives of patients with OFCs (Sivertsen et al., 2008; Grosen et al., 2010), exploring alternative genetic mechanisms beyond simple fetal or maternal gene-effects alone may be important. This entails investigating higher-order interactions, such as epistasis (Cordell, 2009; Wei et al., 2014) and gene-environment interaction (Thomas, 2010), and studying parent-of-origin (PoO) effects (Ferguson-Smith, 2011; Guilmatre and Sharp, 2012; Lawson et al., 2013; Peters, 2014; Connolly and Heron, 2015; Gjerdevik et al., 2017; Haaland et al., 2017).

A PoO effect describes the situation where the effect of an allele in the offspring differs according to the parental origin of the allele (Guilmatre and Sharp, 2012; Gjerdevik et al., 2017). PoO effects are particularly relevant for birth defects because the mother influences the development of the fetus through the action of her own genes and through providing the prenatal environment for the fetus. To estimate PoO effects, one contrasts the frequency of alleles transmitted to an affected offspring from the mother versus the father, and if transmission distortion to the affected offspring is stronger for mothers than fathers (or vice versa), there is evidence of a PoO effect (Weinberg, 1999; Jugessur et al., 2012b; Connolly and Heron, 2015). PoO effects might also occur on the X chromosome. Given the consistently observed excess of females with cleft palate only (CPO) and excess of males with cleft lip with or without cleft palate (CL/P), OFCs are good examples of traits that might be caused by various types of effects on the X chromosome (including PoO effects). Moreover, several genes on the X chromosome are known to cause syndromic forms of clefts, and there is growing evidence that X-linked genes might also contribute to isolated clefts (Jugessur et al., 2012a; Patel et al., 2013; Fonseca et al., 2015; Wise et al., 2016; Skare et al., 2017).

A major shortcoming of most previous GWAS has been the systematic exclusion of SNPs on the X chromosome prior to analysis, even though this chromosome comprises approximately 5% of the human genome and many genotyping platforms do include X-linked SNPs. This has led to only a few analyses of X-linked markers for complex traits in general (Wise et al., 2013), and for OFCs in particular, only two studies have explored PoO effects at the genome-wide level (Shi et al., 2012; Garg et al., 2014), and neither investigated PoO effects on the X chromosome.

With these gaps in mind, we upgraded our R-package Haplin (Gjessing and Lie, 2006) with new functionalities to enable PoO analyses at the genome-wide level, as well as an assessment of statistical power for different statistical models. The current analyses are based on the largest collection of case-parent triads of OFCs to date (Beaty et al., 2010). We implemented a sliding-window approach to haplotype analysis and used two X-inactivation models, one with and one without the assumption of X-inactivation in females (Yang et al., 2011), to explore PoO effects on the X chromosome. In addition, we performed separate analyses on female offspring alone to allow for possible sex-specific differences.

## Materials and methods

### Study populations

Characteristics of the study populations, the genotyping platform, and the quality control criteria used for data cleaning have been detailed elsewhere (Beaty et al., 2010; Shi et al., 2012; Patel et al., 2013; Skare et al., 2017). Briefly, genotyping was performed on an Illumina Human610-Quad® platform and genotypes for 589,945 SNPs (99.56% of the attempted SNPs) were released and later deposited in the Database of Genotypes and Phenotypes (dbGaP; http://www.ncbi.nlm.nih.gov/gap) under study accession ID phs000094.v1.p1. Genotypes for 14,486 X-chromosome SNPs in 1,291 Asian and 1,118 European case-parent triads of isolated clefts were available for the current analyses. For additional data cleaning, we used PLINK (Purcell et al., 2007) to exclude individuals with more than 10% missing genotypes, SNPs with more than 1% missing genotypes, and SNPs with a minor allele frequency (MAF) less than 0.01. Mendelian errors were not assessed during data cleaning; however, SNPs with more than 30 Mendelian errors were excluded at the analysis stage. After data cleaning, 13,180 X-chromosome SNPs were available for the current PoO analyses. Table 1 shows the number of triads according to ethnicity, cleft subtype and child's sex. For each ethnicity, isolated CL/P and isolated CPO triads were analyzed separately.

**Table 1:** Number of case-parent triads according to ethnicity, cleft subtype, and child's sex.

**Ethnicity**	**Cleft category**	**No. of case-parent triads**		
		**Males**	**Females**	**Total**
Asian	CL/P	681	357	1,038
Asian	CPO	100	153	253
Total	CL/P + CPO	781	510	1,291
European	CL/P	536	304	840
European	CPO	131	147	278
Total	CL/P + CPO	667	451	1,118

### Statistical methods

We recently published a new approach to chromosome-wide analysis of X-linked SNPs using the same dataset as here (Skare et al., 2017). In the current paper, we extend the approach to also cover PoO effects on the X chromosome, as implemented in our statistical software Haplin (Gjessing and Lie, 2006). Two types of analyses were performed on the Asian and European samples: (i) Single-marker analyses, where SNPs were analyzed individually, and (ii) haplotype analyses, where up to four SNPs per sliding window were analyzed together. Haplin fits a log-linear model to genotype data from case-parent triads. It reconstructs haplotypes from multiple-SNP data and estimates the relative risk and confidence interval for one or two copies of a target allele or haplotype. Since the expectation-maximization algorithm is implemented in Haplin, incomplete triads can also be used in the analyses after accounting for missing parental genotypes in the maximum likelihood estimation.

For haplotype analysis, we used the HaplinSlide function in Haplin. For additional information on data formats and the HaplinSlide function, see our recent chromosome-wide analysis of X-linked SNPs (Skare et al., 2017), our website at https://people.uib.no/gjessing/genetics/software/haplin/, or the R help pages for Haplin at https://CRAN.R-project.org/package=Haplin.

### Estimating PoO effects on the X chromosome using haplin

Within the maximum-likelihood framework of Haplin, *autosomal* PoO effects are derived essentially by contrasting the relative frequency of the variant allele when transmitted from the mother to the affected child versus the relative frequency of the variant allele when transmitted from the father; there is evidence of a PoO effect if the frequencies differ (Weinberg, 1999; Gjerdevik et al., 2017). Haplin provides estimates of RR_m_ and RR_f_, which are the relative risk increase (or decrease) associated with inheriting the variant allele from the mother and from the father, respectively. The measure of PoO effect is then the ratio of relative risks, RRR = RR_m_/RR_f_, i.e., the PoO effect is a comparison of the relative risks derived from the maternally and paternally inherited alleles. A value of RRR = 1 would be obtained from an allele with no PoO effect, i.e., RR_m_ = RR_f_. Note that even when RRR = 1, RR_m_ and RR_f_ could themselves still be different from 1, meaning that there could be an effect of the allele carried by the fetus even in the absence of a PoO effect. Under a multiplicative model, the risk is assumed to be RR_m_^*^RR_f_ when the allele is inherited both from the mother and the father (i.e., a double dose of the variant allele).

Similarly, on the X chromosome, the estimates of RR_m_ and RR_f_ can be obtained by restricting the analyses to girls only. However, since the X chromosome in boys is maternally derived, Haplin allows an increase in power by combining the relative risk estimate RR_B_ from boys with the estimates from girls. The two options for doing so assume either X-inactivation among girls, where RR_m_^*^RR_f_ = RR_B_, or no X-inactivation, where RR_m_ = RR_B_. Note that in the model assuming X-inactivation, equating the estimate RR_B_ from the boys with the product of RR_m_ and RR_f_ may influence the RR_m_ and RR_f_ estimates themselves, but has relatively little influence on the ratio RRR. When studying the significance of PoO specifically, the hits obtained from investigating girls only will thus be similar to those obtained from the model assuming X-inactivation. The combined model assuming no X-inactivation, however, may well produce other hits. Both combined models allow different baselines risks for boys and girls to be fitted.

**Figure 1:**
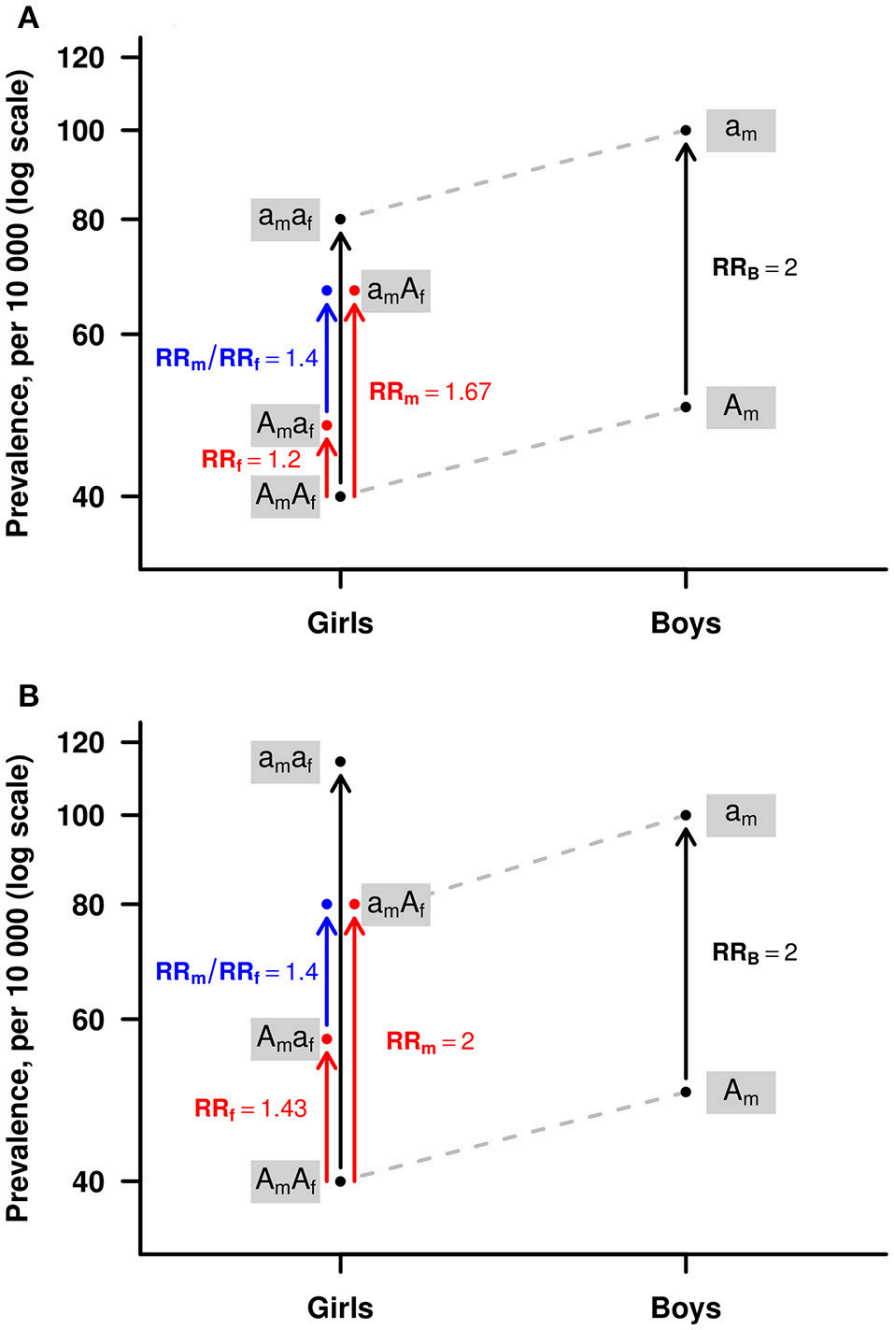
An illustration of the Haplin model for parent-of-origin (PoO) effects on the X chromosome. The red arrows show the relative risks associated with girls inheriting the risk allele “a” from the mother (RR_m_) or from the father (RR_f_). Under the multiplicative risk model illustrated here, the relative risk increase for “aa” girls, i.e., girls inheriting allele “a” from both the mother and the father, is RRaa = RR_m_^*^RR_f_. The ratio RR_m_/RR_f_ = 1.4 is a measure of the PoO effect (blue arrow). The risk increase for boys when inheriting the “a” allele is RR_B_ = 2.0. Under the assumption of X-inactivation **(A)**, the risk increase for girls inheriting “aa” is the same as that for a single “a” in boys, i.e., RRaa = RR_B_. Under the assumption of no X-inactivation **(B)**, the risk increase for girls inheriting “a” from the mother is the same as that for boys, i.e., RR_m_ = RR_B_. In the model without X-inactivation, when a girl inherits “a” from the father as well, this may lead to a higher total risk increase for girls than for boys inheriting the one “a” from the mother. In this illustration, RRaa = RR_m_^*^RR_f_ = 2.86 > RR_B_. The model allows different baseline risks for girls and for boys, here 0.4 and 0.5%, respectively.

Figure 1 provides a detailed explanation of the Haplin model for PoO effects on the X chromosome.

### Post-processing of results

For each cleft subtype, –log_10_(p) were plotted against chromosomal position in a Manhattan plot, before all the Manhattan plots were collated and displayed in a single figure. To control for the proportion of falsely rejected hypotheses, we applied a false discovery rate (FDR) method where the original *p*-values were replaced by “*q*-values” (Storey and Tibshirani, 2003). For example, among SNPs with a *q*-value ≤ 0.2, one would expect an FDR of less than 20%.

### Electronic database information

Haplin version 6.2.1 is implemented as a package in the R statistical software (R Development Core Team, 2014) and can be installed from the CRAN library. More information can be found at our web site (https://people.uib.no/gjessing/genetics/software/haplin/).

### Ethics approval

Ethics approvals for the International Cleft Consortium were obtained from the respective institutional review boards of the participating sites. The consortium was formed in 2007 and each participating institution approved research protocols for the recruitment of case-parent triads from 13 individual sites. All participants have granted their written informed consents. The participating sites included institutions in the US (Johns Hopkins University; University of Iowa; Utah State University; National Institute of Environmental Health Sciences (NIEHS); University of Pittsburgh), Denmark (University of Southern Denmark), Norway (University of Bergen), China (Peking University Health Science Center; Wuhan University; Peking Union Medical College; West China School of Stomatology, Sichuan University; School of Stomatology, Beijing University), Korea (Yonsei University), Taiwan (Chang Gung Memorial Hospital), and Singapore (KK Women's & Children's Hospital; National University of Singapore). For more details on the recruitment sites, the research approvals and protocols, see the online “Supplementary Note” of the original publication (Beaty et al., 2010), as well as the study outline at dbGAP (https://www.ncbi.nlm.nih.gov/gap), under study accession number phs000094.v1.p1.

## Results

**Figure 2:**
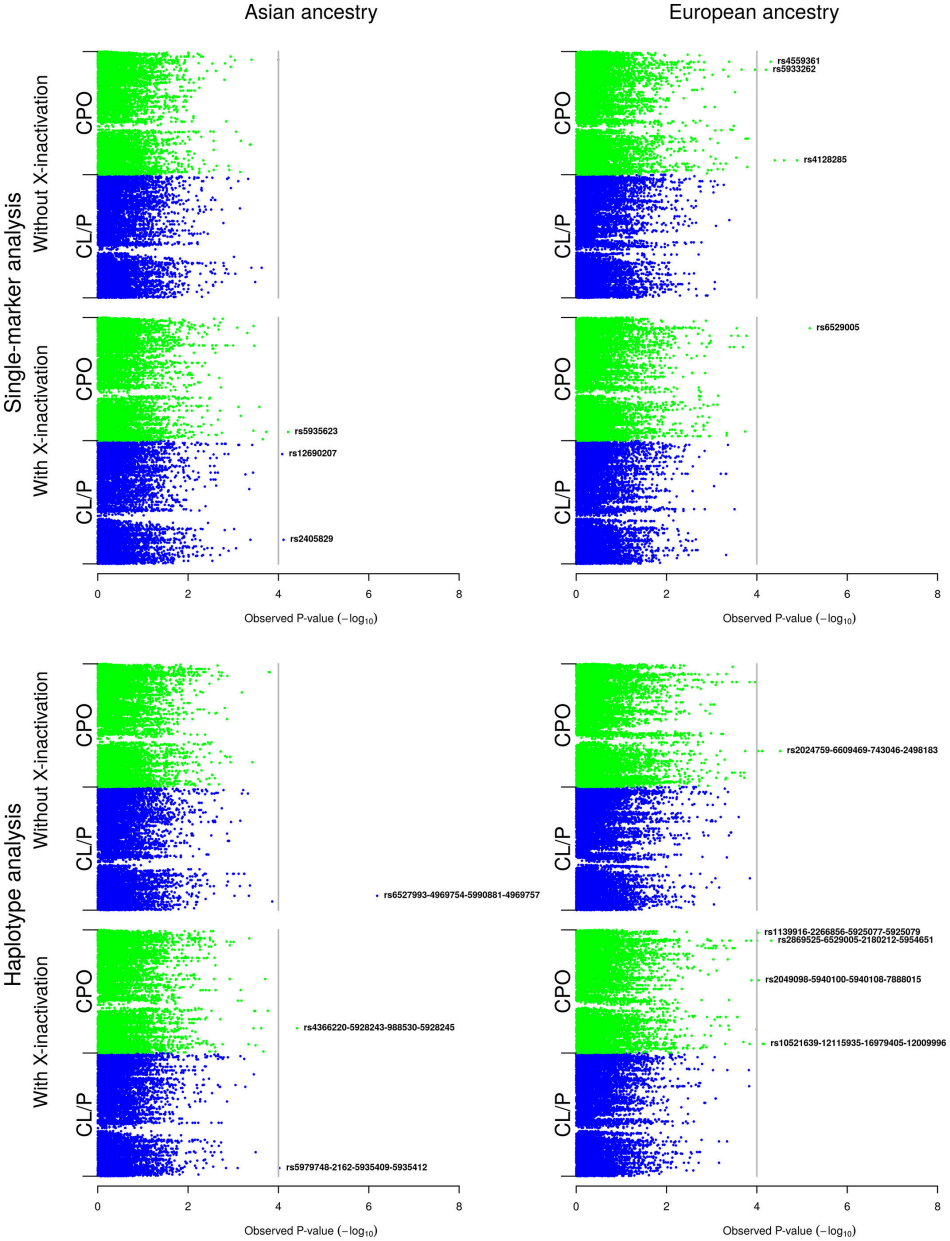
Single-marker and haplotype analyses in the Asian and European samples *without* stratification by child's sex. The Manhattan plots show the single-marker and haplotype analyses based on the model without and with X-inactivation in females, respectively. For convenience, we have added a vertical line corresponding to a Bonferroni-corrected *p*-value cutoff of 10^−4^.

**Figure 3:**
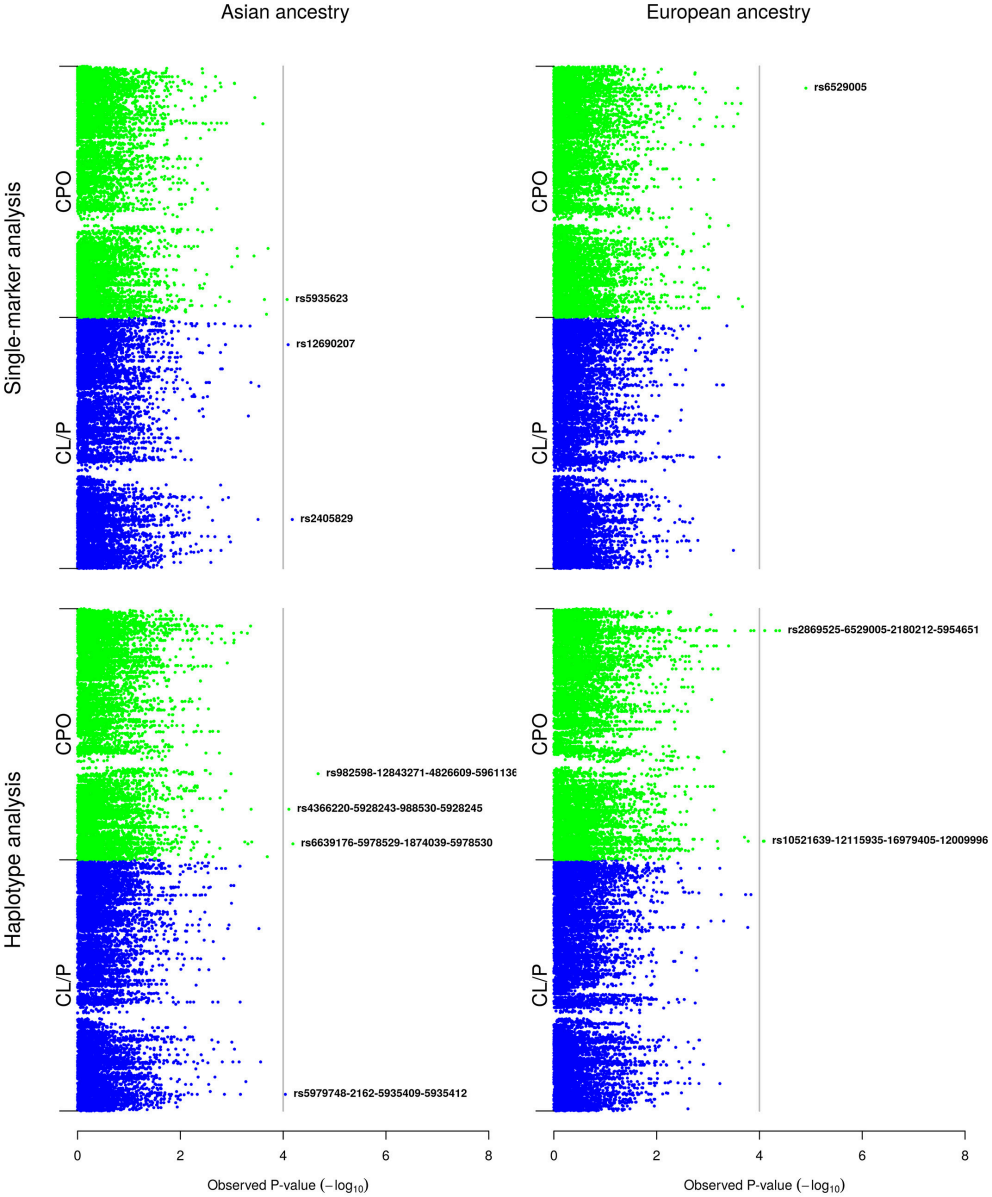
Single-marker and haplotype analyses in the Asian and European girls only. The Manhattan plots show the single-marker and haplotype analyses of the girls only. For convenience, we have added a vertical line corresponding to a Bonferroni-corrected *p*-value cutoff of 10^−4^.

The results are organized in two main parts: (Part A) presents the results of the analyses of the boys and girls together (Figure 2), and (Part B) presents the results of the analyses of the girls only (Figure 3). For each of the X-inactivation models depicted in Figure 2, the results of the single-marker analyses are presented first, followed by the results of the haplotype analyses. Table 2 provides additional information on the SNPs and haplotypes, along with their relative risks (RRs) and 95% confidence intervals (CIs). Note that the Manhattan plots for the single-marker and haplotype analyses (Figures 2, 3) only show the lead SNPs and haplotypes. A more complete list of SNPs and haplotypes lying above the *p*-value cutoff of 10^−4^ in Figures 2, 3 is provided in Table 2.

**Table 2:** *Q*-values and RR estimates for associations identified with SNPs and haplotypes.

**Analysis type**	**Population**	**Model/Sex**	**Isolated cleft type**	**SNP ID**	**Mendelian errors**	***Q*-value**	***P*-value**	**HWE *p*-value**	**Reference allele/haplotype**	**Best haplotype**	**RRm (95% CI)**	**RRm *p*-value**	**RRf (95% CI)**	**RRf *p*-value**	**RRm/RRf (95% CI)**	**RRm/RRf *p*-value**
**ANALYSES BELOW CORRESPOND TO THOSE IN FIGURE** **2**																
Single-marker	Asian	X-inactivation	CPO	rs5935623	0	0.581	6.14e-05	0.54	T	a	1.6 (1.1–2.4)	0.011	0.48 (0.31–0.75)	0.0011	3.4 (1.8–6.2)	8.8e-05
Single-marker	Asian	X-inactivation	CL/P	rs12690207	0	0.471	8.26e-05	0.38	T	a	1.4 (1.1–1.7)	0.00066	0.72 (0.6–0.87)	0.00085	1.9 (1.4–2.6)	7.6e-05
Single-marker	Asian	X-inactivation	CL/P	rs2405829	0	0.471	7.7e-05	0.91	T	a	1.5 (1.2–1.8)	0.00024	0.72 (0.59–0.88)	0.0019	2 (1.4–2.8)	7.1e-05
Single-marker	European	No X-inactivation	CPO	rs4128285	0	0.155	1.29e-05	0.035	A	t	1.5 (1.1–2)	0.017	0.55 (0.36–0.85)	0.0071	2.7 (1.7–4.2)	2.5e-05
Single-marker	European	No X-inactivation	CPO	rs5990877	0	0.155	4.02e-05	0.2	G	c	1.5 (1–2.1)	0.029	0.53 (0.32–0.86)	0.012	2.8 (1.7–4.7)	0.00012
Single-marker	European	No X-inactivation	CPO	rs5950318	0	0.155	2.51e-05	0.074	C	g	1.5 (1.1–2.1)	0.018	0.51 (0.31–0.86)	0.01	2.9 (1.7–5)	7.1e-05
Single-marker	European	No X-inactivation	CPO	rs5933262	0	0.157	6.29e-05	0.3	C	g	1.4 (0.99–1.9)	0.056	0.57 (0.38–0.85)	0.0057	2.4 (1.5–3.7)	8.6e-05
Single-marker	European	No X-inactivation	CPO	rs4559361	0	0.155	4.95e-05	0.91	T	a	1.5 (1.1–2.1)	0.011	0.61 (0.39–0.94)	0.026	2.5 (1.6–3.9)	7.8e-05
Single-marker	European	X-inactivation	CPO	rs6529005	0	0.087	6.74e-06	0.12	T	a	1.6 (1.1–2.3)	0.0084	0.44 (0.29–0.64)	3.00E-05	3.7 (2.1–6.6)	9.00E-06
Haplotype	Asian	No X-inactivation	CL/P	rs6527993-rs4969754-rs5990881-rs4969757	0	0.00814	6.52e-07	0.11	T-A-T-T	g-c-T-T	0.76 (0.48–1.2)	0.23	2 (1.2–3.2)	0.0051	0.38 (0.22–0.66)	0.00051
Haplotype	Asian	X-inactivation	CPO	rs4366220-rs5928243-rs988530-rs5928245	1	0.462	3.88e-05	0.11	G-C-C-C	c-C-C-C	0.45 (0.23–0.93)	0.027	3 (1.6–5.5)	0.00042	0.15 (0.058–0.41)	0.00016
Haplotype	Asian	X-inactivation	CL/P	rs5979748-rs2162-rs5935409-rs5935412	0	0.931	9.53e-05	0.068	A-C-G-T	t-C-G-a	1.4 (1.1–1.7)	0.0056	0.75 (0.59–0.94)	0.014	1.8 (1.2–2.7)	0.0023
Haplotype	European	No X-inactivation	CPO	rs1884299-rs6611365-rs2064596-rs2024759	0	0.236	7.75e-05	0.4	C-T-C-A	C-a-C-t	0.29 (0.033–2.7)	0.27	4.4 (1.1–17)	0.032	0.067 (0.0077–0.57)	0.014
Haplotype	European	No X-inactivation	CPO	rs6611365-rs2064596-rs2024759-rs6609469	0	0.236	9.41e-05	0.4	T-C-A-A	a-C-t-t	1 (0.47–2.2)	0.98	3.7 (1.8–7.5)	0.00037	0.28 (0.13–0.6)	0.0011
Haplotype	European	No X-inactivation	CPO	rs2024759-rs6609469-rs743046-rs2498183	0	0.236	3.05e-05	0.09	A-A-T-T	t-t-T-a	0.66 (0.32–1.4)	0.27	3.6 (1.9–6.8)	0.00011	0.19 (0.088–0.39)	9.6e-06
Haplotype	European	No X-inactivation	CPO	rs743046-rs2498183-rs6651580-rs7050878	0	0.236	7.6e-05	0.09	T-T-C-G	T-a-C-G	0.81 (0.49–1.4)	0.42	3 (1.7–5.3)	0.00011	0.27 (0.15–0.49)	1.8e-05
Haplotype	European	X-inactivation	CPO	rs11798134-rs10521639-rs12115935-rs16979405	1	0.169	7.39e-05	0.18	G-A-T-A	G-t-T-A	2.4 (1.4–4.1)	0.0017	0.43 (0.23–0.78)	0.0064	5.6 (2.3–14)	0.00016
Haplotype	European	X-inactivation	CPO	rs10521639-rs12115935-rs16979405-rs12009996	0	0.169	7.05e-05	0.4	A-T-A-C	t-T-A-C	2.2 (1.3–3.6)	0.0019	0.42 (0.23–0.74)	0.0038	5.3 (2.3–12)	0.00012
Haplotype	European	X-inactivation	CPO	rs2049098-rs5940100-rs5940108-rs7888015	3	0.169	9.14e-05	0.064	C-C-T-c	g-g-T-G	3.5 (1.4–8.7)	0.0064	1.1 (0.47–2.7)	0.79	3.1 (0.77–12)	0.1
Haplotype	European	X-inactivation	CPO	rs2869525-rs6529005-rs2180212-rs5954651	1	0.169	4.81e-05	0.06	T-T-C-A	a-a-g-t	1.7 (1.1–2.6)	0.02	0.38 (0.23–0.63)	0.00013	4.4 (2.1–8.9)	6.1e-05
Haplotype	European	X-inactivation	CPO	rs6529005-rs2180212-rs5954651-rs5907294	1	0.169	9.82e-05	0.031	T-C-A-T	a-g-t-a	1.6 (1.1–2.3)	0.016	0.45 (0.3–0.67)	9.7e-05	3.5 (1.9–6.4)	4.4e-05
Haplotype	European	X-inactivation	CPO	rs1139916-rs2266856-rs5925077-rs5925079	0	0.169	9.63e-05	0.26	T-C-C-T	g-g-g-T	0.14 (0.031–0.67)	0.013	5.6 (1.9–17)	0.0018	0.026 (0.0034–0.19)	0.00038
**ANALYSES BELOW CORRESPOND TO THOSE IN FIGURE** **3**																
Single-marker	Asian	Females	CPO	rs5935623	0	0.564	8.38e-05	0.53	T	a	1.5 (0.88–2.6)	0.14	0.45 (0.24–0.87)	0.015	3.4 (1.8–6.2)	0.00016
Single-marker	Asian	Females	CL/P	rs2405829	0	0.458	6.65e-05	0.49	T	a	1.7 (1.2–2.4)	0.0021	0.84 (0.59–1.2)	0.37	2 (1.4–2.9)	7.9e-05
Single-marker	Asian	Females	CL/P	rs12690207	0	0.458	8.00E-05	0.64	T	a	1.4 (1–1.9)	0.04	0.72 (0.52–1)	0.057	1.9 (1.4–2.7)	8.9e-05
Single-marker	European	Females	CPO	rs6529005	0	0.161	1.25e-05	0.23	T	a	1.5 (0.89–2.4)	0.14	0.42 (0.23–0.77)	0.0044	3.5 (1.9–6.2)	2.5e-05
Haplotype	Asian	Females	CPO	rs982598-rs12843271-rs4826609-rs5961136	0	0.247	2.09e-05	0.57	G-c-a-g	G-G-T-T	0.17 (0.054–0.51)	0.0019	1.7 (0.89–3.2)	0.098	0.097 (0.032–0.3)	4.2e-05
Haplotype	Asian	Females	CPO	rs4366220-rs5928243-rs988530-rs5928245	0	0.304	7.73e-05	0.035	G-C-C-C	c-C-C-C	0.47 (0.14–1.6)	0.22	2.9 (1.2–7.1)	0.02	0.16 (0.057–0.48)	0.00087
Haplotype	Asian	Females	CPO	rs6639176-rs5978529-rs1874039-rs5978530	0	0.304	6.46e-05	0.09	A-C-C-C	A-C-g-C	0.19 (0.039–0.95)	0.044	1.4 (0.52–3.6)	0.52	0.14 (0.03–0.65)	0.012
Haplotype	Asian	Females	CL/P	rs5979748-rs2162-rs5935409-rs5935412	0	0.837	9.07e-05	0.29	A-C-G-T	t-C-G-a	1.6 (1.1–2.4)	0.015	0.86 (0.57–1.3)	0.5	1.9 (1.3–2.8)	0.0021
Haplotype	European	Females	CPO	rs2869525-rs6529005-rs2180212-rs5954651	1	0.195	4.06e-05	0.23	T-T-C-A	a-a-g-t	1.6 (0.83–2.9)	0.16	0.36 (0.17–0.79)	0.0093	4.3 (2–8.9)	0.00014
Haplotype	European	Females	CPO	rs10521639-rs12115935-rs16979405-rs12009996	0	0.195	8.13e-05	0.17	A-T-A-C	t-T-A-C	2.4 (1.1–5)	0.026	0.41 (0.14–1.2)	0.11	5.8 (2.2–16)	5.00E-04
Haplotype	European	Females	CPO	rs11798134-rs10521639-rs12115935-rs16979405	1	0.195	8.39e-05	0.14	G-A-T-A	G-t-T-A	2.2 (0.95–5.2)	0.063	0.39 (0.12–1.2)	0.1	5.8 (2.1–16)	0.00076
Haplotype	European	Females	CPO	rs5954609-rs3135496-rs11095966-rs5954610	0	0.195	4.79e-05	0.23	G-A-A-G	G-t-A-G	0.92 (0.54–1.6)	0.75	0.36 (0.2–0.64)	0.00055	2.5 (1.5–4.5)	0.0013
Haplotype	European	Females	CPO	rs5954635-rs2869525-rs6529005-rs2180212	0	0.195	7.89e-05	0.23	T-T-T-C	a-a-a-g	1.4 (0.75–2.5)	0.29	0.37 (0.18–0.78)	0.0084	3.8 (1.8–7.8)	0.00036

**Figure 4:**
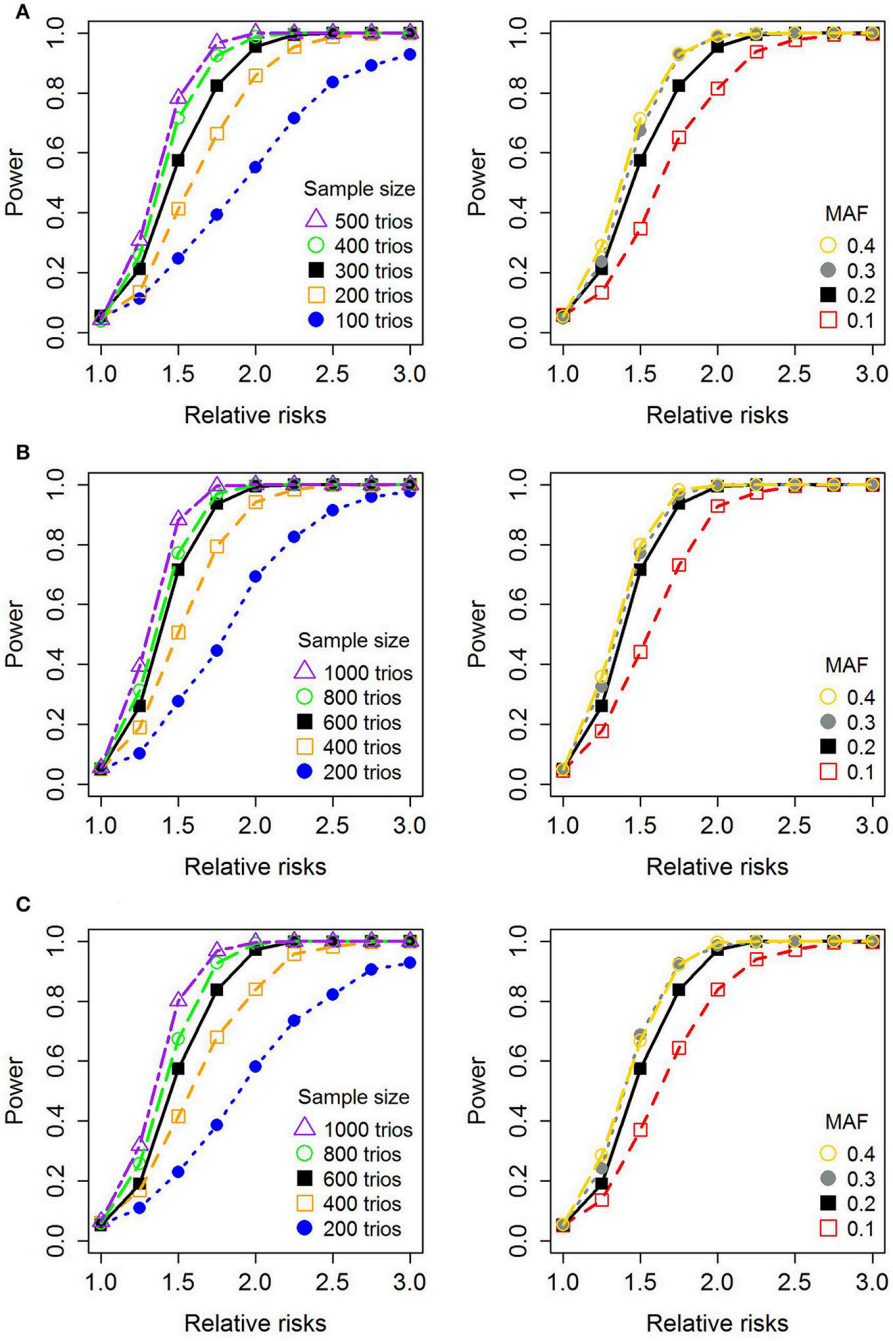
Statistical power for a single SNP. **(A)** Shows the simulated power for the model restricted to girls only, **(B)** shows the simulated power for the model without X-inactivation, and **(C)** shows the simulated power for the model with X-inactivation. The figures on the left depict the power with increasing relative risk ratios (RRRs) (increasing values of RR_m;_ RR_f_ = 1) and differing sample sizes of case-parent triads, using a fixed minor allele frequency (MAF) of 0.2. The figures on the right-hand side show the power with varying RRRs and MAFs, assuming a total of 300 case-parent triads for the girls-only analyses in **(A)** and 600 case-parent triads in **(B,C)** (assuming an equal distribution of boys and girls). We used a nominal significance level of 0.05 throughout.

Figure 4 shows the results of power simulations for different statistical models (girls only, without X-inactivation, with X-inactivation), based on sample sizes reflecting those available in the current GWAS dataset. Table 3 provides a synopsis of all the genes in which associations were identified in the current analyses. To determine whether a given SNP or haplotype was located in or near a gene within 20 kb, we used the 1,000 Genomes browser (https://www.ncbi.nlm.nih.gov/variation/tools/1000genomes).

**Table 3:** Synopsis of the genes in which associations were identified in this study.

**Ethnicity/Statistical model/Sex**	**Type of analysis**	**Gene ID (location)^a^**	**Gene full name**	**Gene description and additional comments^b^**	**Key references**
Asian; X-inactivation	Single-marker	***EGFL6*** **(Xp22.2)**	EGF like domain multiple 6	*EGFL6* is an EGF-repeat containing gene belonging to the EGF-repeat superfamily. Members of this gene family are involved in cell cycle regulation and proliferation. The mouse homolog, *Egf16*, is strongly expressed in the mesenchymal components of both the hard and soft palate from embryonic stage E12.5.	Buchner et al., 2000a,b
		*FGF13* (Xq26.3-q27.1)	Fibroblast growth factor 13	FGF13 belongs to the FGF family of signaling molecules—one of the largest growth factor families. FGF signaling is essential to the development of craniofacial structures. Several FGF ligands and receptors are expressed in the developing facial primordia, and mutations in FGF receptors are known to cause different clefting syndromes (e.g. Kallmann and Apert).	Nie et al., 2006; Rahimov et al., 2012; Du et al., 2016
		*DMD* (Xp21.2-p21.1)	Dystrophin	Dystrophin is a component of the dystrophin-glycoprotein complex whose function is to provide stability to muscle membranes by bridging the cytoskeleton of the muscle to the extracellular matrix. Mutations in *DMD* cause Duchenne (DMD) and Becker (BMD) Muscular Dystrophies. Associations between variants in *DMD* and OFCs have been reported in four chromosome-wide analyses of X-linked SNPs.	Michele and Campbell, 2003; Patel et al., 2013; Fonseca et al., 2015; Wise et al., 2016; Skare et al., 2017
Asian; No X-inactivation	Haplotype	*DMD* (Xp21.2-p21.1)	Dystrophin	As above.	As above.
		***PRPS2*** **(Xp22.2)**	Phosphoribosyl pyrophosphate synthetase 2	This gene encodes a phosphoribosyl pyrophosphate synthetase that plays a central role in protein and nucleotide biosynthesis.	Cunningham et al., 2014
European; No X-inactivation	Single-marker	*SPANXN4* (Xq27.3)	SPANX family member N4	*SPANXN4* belongs to the SPANX multigene family of cancer/testis-specific antigens that play important roles in spermiogenesis.	Kouprina et al., 2004
		*USP26* (Xq26.2)	Ubiquitin specific peptidase 26	USP26 belongs to a large family of deubiquitinating enzymes and this gene is specifically expressed in testis tissue. Variants in *USP26* have been linked to male infertility in some but not all studies.	Stouffs et al., 2005; Wosnitzer et al., 2014; Zhang et al., 2015
European; No X-inactivation	Haplotype	*ZNF157* (Xp11.3)	Zinc finger protein 157	ZNF157 belongs to the zinc-finger family of transcription factors. *ZNF157* is part of a gene cluster of ZNF genes on chromosome Xp11.23.	Derry et al., 1995
		*GABRE* (Xq28)	Gamma-aminobutyric acid type A receptor epsilon subunit	*GABRE* encodes the “Gamma-aminobutyric acid type A receptor epsilon subunit.” This gene maps to a cluster on chromosome Xq28 that houses other subunits of the same receptor. Fatemi et al. (2013) reported increased expression of *GABRE* in the lateral cerebella of subjects with schizophrenia, bipolar disorder and major depressive disorder.	Fatemi et al., 2013
Asian; Girls only	Single-marker	***EGFL6*** **(Xp22.2)**	EGF like domain multiple 6	As above.	As above.
		*FGF13* (Xq26.3-q27.1)	Fibroblast growth factor 13	As above.	As above.
		*DMD* (Xp21.2-p21.1)	Dystrophin	As above.	As above.
Asian; Girls only	Haplotype	*ITIH6* (Xp11.22)	Inter-alpha-trypsin inhibitor heavy chain family member 6	*ITIH6* encodes a protein belonging to the inter-alpha trypsin inhibitor heavy chain (ITIH) family. Al-Mubarak et al. (2017) performed whole-exome sequencing of autism spectrum disorders (ASD) triads and uncovered rare variants in several X-linked genes, including *ITIH6*.	Al-Mubarak et al., 2017
		***FRMPD4*** **(Xp22.2)**	FERM and PDZ domain containing 4	*FRMPD4* encodes a multi-domain (PDZ and FERM) containing protein. It modulates the activity of key postsynaptic scaffold proteins that are involved in cognitive processes. Variants in *FRMPD4* have been associated with X-linked intellectual disability and schizophrenia.	Hu et al., 2016; Matosin et al., 2016; Trujillano et al., 2017
		***PRPS2*** **(Xp22.2)**	Phosphoribosyl pyrophosphate synthetase 2	As above.	As above.

a *Genes located within the same chromosomal band Xp22.2 are emboldened*.

b *Information on these genes was collated from the NCBI Entrez gene database (https://www.ncbi.nlm.nih.gov/gene) and the references shown*.

### (A) Single-marker and haplotype analyses combining estimates from boys and girls

Figure 2 displays the results of the single-marker and haplotype analyses in the Asian and European samples for each X-inactivation model.

#### (i) Asian sample

There were no associations in the single-marker analyses of isolated CPO or isolated CL/P in the model without X-inactivation. By contrast, three SNPs stood out in the model with X-inactivation: rs5935623 in CPO; and both rs12690207 and rs2405829 in CL/P. rs5935623 is located in the gene for “EGF-like domain multiple 6” (*EGFL6* at Xp22.2), rs12690207 is located in “Fibroblast growth factor 13” (*FGF13* at Xq26.3-q27.1), and rs2405829 is located in “Dystrophin” (*DMD* at Xp21.2-p21.1).

In haplotype analyses, haplotype rs6527993-rs4969754-rs5990881-rs4969757 was associated with CL/P in the model without X-inactivation. Although the q-value for this haplotype was 0.008 (Table 2), none of the SNPs is located in or near a gene within 20 kb. In the model with X-inactivation, we found associations with two haplotypes: (i) rs4366220-rs5928243-rs988530-rs5928245 in CPO and (ii) rs5979748-rs2162-rs5935409-rs5935412 in CL/P. The first SNP in haplotype (i) lies ~9.7 kb from *DMD*, and the first SNP in haplotype (ii) lies ~5.3 kb from “Phosphoribosyl pyrophosphate synthetase 2” (*PRPS2* at Xp22.2).

#### (ii) European sample

In the model without X-inactivation, we found associations with rs4559361, rs5933262, and rs4128285 in CPO (all three SNPs had the same *q*-value of 0.155; Figure 2, Table 2). rs4559361 lies ~4.4 kb from the gene for “SPANX family, member N4” (*SPANXN4* at Xq27.3), rs5933262 is located in “Ubiquitin specific peptidase 26” (*USP26* at Xq26.2), and rs4128285 is not located near any gene within 20 kb. In the model with X-inactivation, rs6529005 was associated with CPO. This SNP is not located near any gene within 20 kb.

In haplotype analyses, rs2024759-rs6609469-rs743046-rs2498183 was associated with CPO in the model without X-inactivation. The last SNP in this haplotype is located ~24.2 kb from “Zinc finger protein 157” (*ZNF157* at Xp11.3). In the model with X-inactivation, four haplotypes were associated with CPO, but only rs1139916-rs2266856-rs5925077-rs5925079 (*q* = 0.169) has SNPs located in a specific gene—in “Gamma-aminobutyric acid type A receptor epsilon subunit” (*GABRE* at Xq28).

### (B) Single-marker and haplotype analyses restricted to girls only

Figure 3 displays the results of the single-marker and haplotype analyses restricted to the Asian and European girls only.

#### (i) Asian sample

In single-marker analyses, we identified associations with the same three SNPs (rs5935623 in CPO, and both rs12690207 and rs2405829 in CL/P) as in the single-marker analyses based on the model with X-inactivation (Figure 2). In the haplotype analyses, two of the haplotypes that were associated in the analyses based on the model with X-inactivation (Figure 2) were also identified here and in the same cleft categories; notably, rs4366220-rs5928243-rs988530-rs5928245 in CPO and rs5979748-rs2162-rs5935409-rs5935412 in CL/P. There were two additional haplotypes associated with CPO: (i) rs982598-rs12843271-rs4826609-rs5961136 and (ii) rs6639176-rs5978529-rs1874039-rs5978530. The last SNP in haplotype (i) is located in “Inter-alpha-trypsin inhibitor heavy chain family member 6” (*ITIH6* at Xp11.22), while the SNPs in haplotype (ii) are located in “FERM and PDZ domain containing 4” (*FRMPD4* at Xp22.2).

#### (ii) European sample

In single-marker analyses, rs6529005 (*q* = 0.161) was associated with CPO, which was also identified in the single-marker analyses in the same cleft category based on the model with X-inactivation (Figure 2). There were no associations with CL/P. In the haplotype analyses, the same SNP identified in the single-marker analysis above, rs6529005, appeared to be driving the association here between rs2869525-rs6529005-rs2180212-rs5954651 and CPO. One more haplotype was associated with CPO: rs10521639-rs12115935-rs16979405-rs12009996. Both of these haplotypes were already identified in the haplotype analyses based on the model with X-inactivation (Figure 2), but none of the SNPs in these haplotypes is located near any known gene within 20 kb.

### Power simulations

Haplin includes a complete framework for power simulations (Gjerdevik et al., 2017). Figure 4 displays the *a priori* power calculations for a single SNP to detect PoO effects on the X chromosome. All calculations were based on 1,000 simulated datasets with a 0.05 nominal significance level (note that a baseline risk of one was used throughout). The power of a single SNP to detect PoO effects on the X chromosome depends on several factors, including the minor allele frequency (MAF), effect size, sample size and family design. The power of the model focusing on girls only is similar to that of the model with X-inactivation in females (Figures 4A,C), which is consistent with the fact that those models provide similar estimates of RRR (as also mentioned in the Materials and Methods section). Overall, the power is sufficient for RRRs > 2, with the range of MAFs and sample sizes presented in our analyses (RRRs ≥ 2.5 for the smallest sample sizes).

## Discussion

Our analyses detected possible PoO effects with several SNPs and haplotypes on the X chromosome, some of which were located in or close to genes (summarized in Table 3). Perhaps the most prominent gene on the list is “Dystrophin” (*DMD*), the second largest gene in humans according to the size of the transcript and protein product (Richards and Hawley, 2011). Dystrophin forms part of the dystrophin-glycoprotein complex whose function is to provide mechanical stability to the plasma membrane of striated muscle cells (Michele and Campbell, 2003; Gao and McNally, 2015). Several lines of evidence point to a link between muscular dystrophy and OFCs. For example, both congenital muscular dystrophy and CL/P have been observed in the rare autosomal-recessive Walker-Warburg syndrome (Moore et al., 1988; Dobyns et al., 1989; Pratap et al., 2007; Vajsar et al., 2008). More recently, we and others have reported associations between OFCs and variants in *DMD* in several chromosome-wide studies of X-linked SNPs (Patel et al., 2013; Fonseca et al., 2015; Wise et al., 2016; Skare et al., 2017). Even though only the Fonseca et al. (2015) study was based on a different GWAS dataset and study population compared to the other three studies, the repeated identification of *DMD* nevertheless constitutes an exciting new direction to pursue in future OFCs research.

Besides *DMD*, we identified PoO effects with several other genes, two of which—*EGFL6* and *FGF13*—offer biologically plausible links to OFCs. *EGFL6* belongs to the EGF-repeat containing superfamily of genes known to be involved in cell cycle regulation and proliferation (Buchner et al., 2000a). Expression analyses of murine *Egf16* show strong expression in the mesenchymal components of both the hard and soft palate from embryonic stage E12.5 (Buchner et al., 2000b). This spatio-temporal pattern of gene expression supports a role for *EGFL6* in the development of the orofacial complex, but as with *DMD*, the associations with *EGFL6* will need to be validated in other OFCs samples.

FGF13 belongs to the FGF family of signaling molecules known to play key roles in embryonic development (Nie et al., 2006; Rahimov et al., 2012; Du et al., 2016). Several FGF ligands and receptors are expressed in the developing facial primordia, and mutations in specific FGF receptors have been reported to cause clefting syndromes (Rahimov et al., 2012). For example, Kallmann syndrome is caused by mutations in *FGFR1* (Dodé et al., 2003) whereas Apert syndrome is caused by mutations in *FGFR2* (Wilkie et al., 1995). *FGF13* is thus a promising gene to pursue in other independent cleft samples.

**Figure 5:**
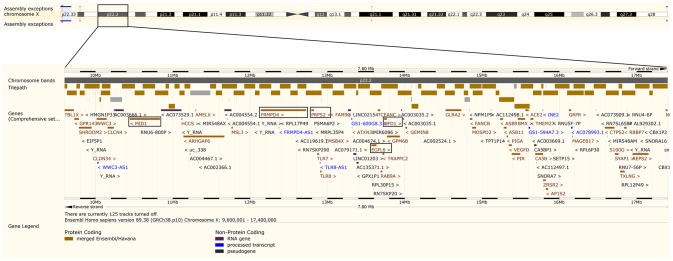
Genes located in chromosomal region Xp22.2. This collage of screenshots of chromosomal region Xp22.2 was generated using the *Ensembl* genome browser (http://www.ensembl.org/) (Kersey et al., 2016; Yates et al., 2016). The three genes identified in this study are shown in boxes, in addition to the “Oral-facial-digital syndrome 1” (*OFD1*) gene, in which we had identified associations in our previous study on candidate genes on the X chromosome (Jugessur et al., 2012a), and the “Midline 1” (*MID1*) gene.

As opposed to the above genes, there are no obvious connections between OFCs and the remaining genes in Table 3. It is nonetheless noteworthy that both *PRPS2* and *FRMPD4* are located in chromosomal region Xp22.2, which is also the location of *EGFL6* whose link to OFCs was discussed above. Figure 5 is a collage of screenshots showing all the genes in chromosomal region Xp22.2 as they appear in the genome browser of The *Ensembl* Project (Kersey et al., 2016; Yates et al., 2016). This chromosomal region contains two genes that have been linked to clefting syndromes. The first is “Oral-facial-digital syndrome 1” (*OFD1*), a gene in which we had previously identified associations with OFCs in a candidate-gene analysis of X-linked markers (Jugessur et al., 2012a). Mutations in *OFD1* underlie the X-linked dominant oral-facial digital syndrome type 1, which is characterized by malformations of the face, oral cavity and digits, and lethality in most affected males (Ferrante et al., 2001). The orofacial abnormalities in Oral-facial-digital syndrome 1 include median cleft lip, clefts of the alveolar ridge, and cleft palate. The second gene in chromosomal region Xp22.2 region is “Midline 1” (*MID1*). Mutations in this gene cause the X-linked Opitz GBBB syndrome, a congenital midline malformation syndrome featuring clefting of the lip and palate as part of the overall clinical picture (Quaderi et al., 1997). Furthermore, associations between isolated CL/P and specific haplotypes in *MID1* have been reported in an Italian population (Scapoli et al., 2008).

As seen in Figure 5, *MID1, FRMPD4, PRPS2, EGFL6*, and *OFD1* all lie relatively close to one another on chromosome Xp22.2. Specifically, *MID1* is ~1.3 Mb from *FRMPD4, FRMPD4* is ~66.8 kb from *PRPS2, PRPS2* is ~745.3 kb from *EGFL6*, and *EGFL6* is ~81.8 kb from *OFD1*. Aside from the potential contributions of the genes themselves, their shared location on Xp22.2 suggests that there might be a specific locus (or loci) in that region driving the associations detected by our PoO analyses—a hypothesis worth pursuing in other OFCs samples. In addition, the apparent excess of genes on the X chromosome that are associated with sexually dimorphic traits (Saifi and Chandra, 1999; Vawter et al., 2004; Berletch et al., 2011) makes genes that escape X-inactivation (Yang et al., 2011; Deng et al., 2014; Balaton and Brown, 2016) particularly attractive candidates in explaining some of the observed male-female differences in development and physiology (Ober et al., 2008; Berletch et al., 2011). Such a scenario would be captured by our model assuming no X-inactivation (Figure 1B). Since both CL/P and CPO consistently exhibit a skewed sex ratio in prevalence, it may be worth exploring this hypothesis in future studies of PoO effects in OFCs. In addition, scenarios involving variable escape from X-chromosome inactivation (Peeters et al., 2014) are also amenable to testing using the X-inactivation models in Haplin.

Identifying associations between isolated OFCs and genes for clefting syndromes is not surprising, as it has long been recognized that Mendelian forms of clefting that phenocopy isolated clefts may provide important clues in identifying genes involved in isolated forms of clefts (Jugessur and Murray, 2005). One of the best examples to date is Van der Woude syndrome (VWS), in which hypodontia and lip pits are the only additional features distinguishing patients with VWS from those with isolated clefts. Mutations in interferon regulatory factor 6 (*IRF6*) were reported to cause VWS (Kondo et al., 2002), and, subsequently, we and others reported associations between *IRF6* variants and isolated clefts (Zucchero et al., 2004; Jugessur et al., 2008, 2009b; Beaty et al., 2013; Leslie et al., 2016b; Moreno Uribe et al., 2017). Our current identification of several genes associated with clefting syndromes, while studying *isolated* OFCs *per se*, supports the notion that there may be a wider spectrum of subclinical features beside the overt cleft itself, blurring the distinction between isolated and non-isolated clefts.

Indeed, a handful of subclinical features have been characterized in patients who were initially classified as “isolated” cleft cases, as well as in their immediate family members (Weinberg et al., 2006; Jugessur et al., 2009a; Marazita, 2012). The inclusion of one such subclinical feature—breaks in the *orbicularis oris* muscle in the mouth—revealed a pattern of familial segregation more akin to that of a Mendelian trait (Marazita, 2007; Neiswanger et al., 2007). This further supports the existence of a wider array of subphenotypes that are associated with the overt cleft, and the identification of genes underlying syndromic clefts while analyzing isolated OFCs samples, like we do in this paper, is consistent with previous descriptions of an “extended cleft phenotype” (Weinberg et al., 2006; Marazita, 2007, 2012; Jugessur et al., 2009a; Rahimov et al., 2012).

Another important factor to consider in genetic association analyses is whether associations identified in one ethnicity are generalizable to other ethnicities. *DMD* was the only gene in which associations were identified in both CPO and CL/P, in the model with X-inactivation, in both single-marker and haplotype analyses, and in Europeans only. The remaining associations were highly specific for ethnicity, cleft subtype and X-inactivation model, with none of the associations in the Asian sample overlapping with those in the European sample. For example, the associations with *EGFL6, FGF13, DMD*, and *PRPS2* were only detected in the Asian sample and in the model with X-inactivation, whereas the associations with *SPANXN4, ZNF157*, and *GABRE* were only observed in the European sample. Furthermore, associations with *SPANXN4* and *ZNF157* were only detected in the model assuming no X-inactivation, whereas associations with *GABRE* were only detected in the model with X-inactivation. These findings challenge the common assumption that the remarkable phenotypic consistency of OFCs across different ethnicities is because at least a subset of the causal variants is shared across different ethnicities. The apparent lack of overlap between Asians and Europeans was also observed in our recent chromosome-wide analysis of X-linked variants in the same GWAS dataset as here (Skare et al., 2017). Collectively, these findings underscore the importance of examining different ethnicities, X-inactivation models and cleft subtypes, even though this means adding more multiplicity to the analyses.

We observed a substantial overlap in the associations identified in the analyses based on the model with X-inactivation (Figure 2) and those identified in the analyses restricted to girls only (Figure 3). This was as expected, for the reasons provided in the Materials and Methods section. Regarding statistical power, our simulations indicated that there would be sufficient power for single-SNP analyses based on relative risk ratios (RRR) ≥ 2 (RRR ≥ 2.5 for the smaller sample of CPO triads) (Figure 4). It could be argued that the assumption of a RRR ≥ 2 or RRR ≥ 2.5 is optimistic in the context of OFCs, or most other complex traits for that matter. Our current analyses of X-linked PoO effects are novel and should be regarded as exploratory at this stage. With continuously accruing sample sizes of OFCs through multiple international collaborations, we envisage our new methods to gain wider currency in future analyses of X-linked PoO-effects in the larger pooled datasets.

This study benefited from being based on the largest collection of case-parent triads of OFCs to date, which enabled two major isolated cleft subtypes—CPO and CL/P—to be analyzed separately and without biases due to population stratification. In addition, having access to genotype data on two major ethnicities (Asian and European) enabled a more in-depth exploration of ethnicity- and sex-specific differences for each of the identified associations. The new methods presented here enabled an investigation of the hitherto untested possibility of PoO effects on the X chromosome, through the use of models with or without the assumption of X-inactivation in females. Limitations of the study include a lack of control triads to verify potential population transmission ratio distortions that could affect the PoO estimates. This could be the case if a genetic variant can cause early abortions that are not even registered as births. However, since the PoO RRR estimates are a ratio of two transmission distortions (from the mother and from the father), distortions unrelated to clefting, such as early abortions, would at least need to exhibit a parent-of-origin dependent pattern to substantially bias the RRR value. Furthermore, the lack of a comparable replication cohort precluded a formal validation of the current findings, which currently makes it difficult to interpret their relevance to clinical research.

To summarize, we developed new methods for a robust investigation of PoO-effects on the entire X chromosome and present the first results of such a screening in the largest available collection of OFCs triads to date. Our analyses identified associations with several genes, in particular with *DMD, FGF13*, and *EFG16* that offer biologically plausible links to OFCs. The additional identification of associations with other genes on chromosomal region Xp22.2 deserves further scrutiny because several genes for clefting syndromes appear to cluster in this region. Except for *DMD*, in which associations were identified in both CPO and CL/P, the rest of the associations were highly specific for ethnicity, cleft subtype and X-inactivation model, highlighting the importance of performing such subanalyses, despite the need to adjust for additional multiple testing. More generally, the new methodology presented here is easily adaptable to the study of X-linked PoO effects in other outcomes that use a family-based study design.

## Author contributions

Conception of the work, study design, data analysis and interpretation, manuscript preparation and final manuscript approval: AJ, ØS, HG, RL, ØH, MG, and JR. Statistical modeling and software design: HG, ØS, MG, JR, and ØH. Funding acquisition: AJ, HG, and RL.

### Conflict of interest statement

The authors declare that the research was conducted in the absence of any commercial or financial relationships that could be construed as a potential conflict of interest.
